# Whole-Body Magnetic Resonance Imaging Assessment of Joint Inflammation in Rheumatoid Arthritis—Agreement With Ultrasonography and Clinical Evaluation

**DOI:** 10.3389/fmed.2020.00285

**Published:** 2020-06-19

**Authors:** Sin Ngai Ng, Mette B. Axelsen, Mikkel Østergaard, Susanne Juhl Pedersen, Iris Eshed, Merete L. Hetland, Jakob M. Møller, Lene Terslev

**Affiliations:** ^1^Copenhagen Center for Arthritis Research, Center for Rheumatology and Spine Diseases, Rigshospitalet, Glostrup, Denmark; ^2^Department of Medicine, Queen Elizabeth Hospital, Kowloon, Hong Kong; ^3^Department of Clinical Medicine, Faculty of Health Sciences, University of Copenhagen, Copenhagen, Denmark; ^4^Department of Diagnostic Imaging, Sheba Medical Center, Tel Giborim Affiliated With Tel Aviv University, Tel Aviv, Israel; ^5^Department of Radiology, Herlev-Gentofte Hospital, Copenhagen, Denmark

**Keywords:** ultrasound, WBMRI, rheumatoid arhtritis, inflammation, agreement

## Abstract

**Objective:** To compare joint inflammation seen by whole-body magnetic resonance imaging (WBMRI), with “whole-body” ultrasound and clinical assessments, in patients with active rheumatoid arthritis (RA) before and during tumor necrosis factor-inhibitor (TNF-I, adalimumab) treatment.

**Methods:** In 18 patients with RA, clinical assessment for joint tenderness and swelling, WBMRI, and ultrasound were obtained at baseline and week 16. Wrist, metacarpophalangeal (MCP) and proximal interphalangeal (PIP), elbow (except for WBMRI), shoulder, knee, ankle, and metatarsophalangeal joints were examined. Joint inflammation was defined by WBMRI as the presence of synovitis and/or osteitis and by ultrasound as gray-scale synovial hypertrophy grade >2 and/or color Doppler grade >1. On patient level, agreement was assessed by Spearman correlation coefficients (rho) for sum scores for 28 joints (i.e., wrists, MCPs, PIPs, elbows, shoulders, and knees) between clinical examination (DAS28CRP), ultrasound (US28), and WBMRI (WBMRI26; elbows not included). On joint level, agreement on inflammation between WBMRI, ultrasound, and clinical findings was calculated with Cohen's kappa (κ).

**Results:** At patient level, WBMRI26 and US28 sum scores showed good correlation (rho = 0.72; *p* < 0.01) at baseline, but not at follow-up (rho = 0.25; *p* = 0.41). At joint level, moderate agreement was seen for hand joints (κ = 0.41–0.44); for other joints κ <0.40. No correlation with DAS28CRP was seen. No statistically significant correlations were observed between changes in WBMRI26, US28, and DAS28CRP during treatment.

**Conclusions:** WBMRI and ultrasound joint inflammation sum scores at patient level showed good agreement in clinically active RA patients before TNF-I initiation, whereas agreement was poorer at joint level, and after treatment.

## Introduction

Suppression of joint inflammation is essential in modern management of rheumatoid arthritis (RA) and is a key element in clinical trials (1, 2) and is traditionally assessed by clinical joint examination, but magnetic resonance imaging (MRI) and ultrasound have been demonstrated to be more sensitive than clinical assessment for detecting joint involvement (3–6) and have been shown to be sensitive to change during treatment with TNF inhibitors (7–10). While, conventional MRI is limited to assessing one or a few joint regions per examination whole-body (WB) MRI has been introduced as a potential method for accurately assessing joint inflammation in the entire body in one session, covering both axial and peripheral joints. Its potential use for monitoring disease activity has been indicated in studies demonstrating a decrease in inflammation scores after biologic treatment in RA (11, 12), psoriatic arthritis (13), and axial spondyloarthritis (14); however, the sensitivity has not been assessed.

Ultrasound can assess multiple joints in one session and several studies have shown that ultrasound has good agreement with conventional MRI for detecting synovitis (3, 4) and is, consequently, a well-suited comparator for the ability of WBMRI for detecting joint inflammation.

The aim of the current study was to assess the agreement between WBMRI findings of joint inflammation with “whole-body” ultrasound joint inflammation and clinical joint assessment and the ability to assess change during treatment with adalimumab in a cohort of clinically active RA patients.

## Methods

### Study Design

The current study was undertaken as a sub-study related to an investigator-initiated clinical trial (EudraCT number NCT01029613), of 37 patients with clinically active (DAS28CRP>3.2) RA, fulfilling the 1987 American College of Rheumatology classification criteria for RA (15) with the aim to use WBMRI to visualize inflammation and structural lesions during treatment with Adalimumab (see Axelsen et al. (11) for details). The patients had to be naïve to biological therapy and initiated treatment with adalimumab 40 mg sub-cutaneous every other week. The patients were not allowed to receive glucocorticoids or any synthetic Disease Modifying antirheumatic Drugs other than methotrexate from 4 weeks before inclusion and throughout the study. The patients included in the main study were invited to participate in the sub-study and 19 of these patients accepted to participate. However, one patient were subsequently excluded due technical problems with the baseline WBMRI.

At each clinical visit bilateral wrist, metacarpophalangeal joints (MCPs) 1–5, proximal interphalangeal joints (PIPs) 1–5, elbow, shoulder and knee joints, ankles, and metatarsal-phalangeal joints (MTPs) 1–5 were assessed for swelling and tenderness. Visual analog scale (VAS, 0–100 mm) assessments of pain and patients and physician's global assessment, Health Assessment Questionnaire (HAQ), and C-reactive protein (CRP) were determined, and the DAS28CRP was calculated. The ultrasound examination was performed prior to the WBMRI with an average of 2 days in between. The clinical examination, the ultrasound examination, and WBMRI were performed at baseline before initiation of treatment and at week 16.

All the patients were seen by the same clinician throughout the study. At 16 weeks, the clinical response was evaluated applying the EULAR response criteria.

The study was approved by the local ethical committee and the Danish Medicines Agency, following the Helsinki Declaration and the Good Clinical Practice guidelines.

### MRI Methodology

All WBMRI scans were performed in the same 3T MRI unit (Achieva, Philips, Best, the Netherlands). Short tau inversion recovery (STIR) and pre-contrast T1-weighted spin-echo images were obtained for six imaging stations, assessing the following anatomical areas: cervical spine, shoulder/thoracic spine, lumbar spine, hips/hands, knees, and feet. The field of view was 470 × 253–287 mm, slice thickness 3 mm for hips/hands and feet, while 5 mm for the other locations. The T1-weighted sequences of hips/hands and feet were repeated after intravenous gadolinium-contrast injection (16).

Joints within the field of view, were read and scored separately for the presence/absence of synovitis and bone marrow edema (BME), respectively, using the validated OMERACT definitions developed for conventional MRI (17).

The examined joints included 26 of the 28 peripheral joints used in DAS28 (elbows were not examined by WBMRI as they were outside the field of view). In addition, ankles metatarsophalangeal joint 1–5 were examined bilaterally. MRI synovitis and BME were separately scored as present/absent (0–1) applying the aforementioned OMERACT definitions and an WBMRI joint inflammation score (range 0–2) was calculated per joint. To assess the inflammation at patient level the score per joint was used for calculating total WBMRI scores per patient (WBMRI26; range 0–52) by summing up the joint score in 26 joints. At joint level, joint inflammation was considered present if either synovitis or BME was present.

The WBMRIs were evaluated by one experienced WBMRI radiologist (IE), who was blinded to time point, clinical and biochemical data. Average duration of the WBMRI examination was 60 min and with similar average duration for evaluation and scoring the WBMRI.

### Ultrasound Methodology

All ultrasound examinations were performed with a General Electric Logiq 9 ultrasound machine equipped with a high-frequency linear probe ML 6–15 MHz. Doppler setting was adjusted for slow flow according to published recommendations (18). The examined joints were the same as for MRI plus the elbows.

Applying the validated OMERACT definition for synovitis (19) all joints were scored using a semi-quantitative score (0–3) for gray scale (GS) and color Doppler (CD) (20). Each component (GS and CD, respectively) was scored separately and subsequently converted to a binary score (presence/absence, 0–1, as follows: positive GS synovitis was defined as a score >2, and positive CD was defined as a score >*1*. Based on these binary scores for GS synovitis and CD an ultrasound joint inflammation score (range 0–2) was calculated per joint, and to assess the inflammation on patient level the joint scores for 28 joints were added to calculate a total US inflammation score per patient (US28 score; range 0–56). At joint level, joint inflammation was considered present if either GS synovitis (>2) or CD (>1) was present. All ultrasound examinations were performed by one experienced sonographer (LT) blinded to clinical and biochemical data, but not to time point. Each ultrasound examination and scoring of the joints for joint inflammation lasted ~60 min.

### Assessment of Agreement at Joint Level

At joint level, the agreement between WBMRI and ultrasound was evaluated using presence vs absence of joint inflammation for the wrists, MCP and PIP 1–5, elbows, shoulders, knees, ankles, and MTP 1–5. In addition, the agreement between clinical SJ and TJ and WBMRI and ultrasound, respectively, was assessed on data from baseline and week16 follow-up, i.e., data from both baseline and follow-up were pooled and analyzed together.

### Assessment of Agreement at Patient Level

To assess the total inflammatory burden at patient level, composite scores were established including only the joints necessary to establish DAS28CRP (wrists, MCP, and PIP1–5, elbows, shoulders, knees). For clinical assessment a DAS28CRP were calculated and 28 tender (TJC28) and 28 swollen (SJC28) joint counts. For ultrasound and MRI the US28 and MRI26 (described above) were used. The correlation between US28 and WBMRI26 and with DAS28CRP was assessed as were the correlation to TJC28 and SJC28.

### Statistics

Agreement between clinical assessment, ultrasound, and WBMRI at joint level was assessed with Cohen's kappa (κ) where κ values 0–0.20 indicates slight, 0.21–0.40 fair, 0.41–0.60 moderate, 0.61–0.80 substantial, and 0.81–1 perfect agreement) (21). Percentages of observed agreement (i.e., percentage of observations that obtained the same score) were also calculated. At patient level, sum scores were compared using the Spearman correlation analyses (rho). *P* < 0.05 was considered statistically significant. Statistical analyses were performed by IBM SPSS program version 20.0 (SPSS Incorporated, Chicago, USA).

## Results

### Clinical Characteristics

Baseline characteristics are shown in Table 1. Eighteen patients were included in the study; 89% women, median age 54.4 years (range 26–73), and median disease duration 4.5 years (range 1–28). Thirteen patients were seen at 16 week-follow-up, whereas 5 were lost to follow up due to lack of treatment effect (2 patients), side effects to medication (1 patient), fracture (1 patient), and patient's cancellation of ultrasound appointment (1 patient).

**Table 1:** Patient characteristics at baseline and follow up.

***n***	**Baseline**	**Week 16**
	**18 patients**	**13 patients**
Gender (female) (%)	89%	85%
Age (years)	54.5 (26–73)	55 (29–73)
Disease duration	4.5 (1–28)	5.5 (1–28)
DAS28 CRP (mg/dl)	4.52 (3.48–6.66)	3.26 (1.97–4.76)
Tender joint count (0–28)	6.5 (2–19)	3 (0–11)
Swollen Joint (0–28)	5.5 (1–13)	1 (0–5)
WBMRI26 inflammation (0–52)	8 (0–26)	5 (1–21)
US28 inflammation (0–56)	4 (0–29)	3 (0–26)

*Values are median (range) if not otherwise indicated. N, number of patients; WBMRI26, whole body magnetic resonance imaging sum score for 26 joints; US28, ultrasound sum score for 28 joints*.

Overall, the cohort had low inflammatory activity at baseline at patient level by both US28 and WBMRI26 with a median (range) US28 score of 4 (0–29) and a WBMRI26 score of 8 (0–26).

### Correlation at Patient Level at Baseline and Follow-Up

The correlation between WBMRI26 and US28 was good at baseline (rho = 0.78; *p* < 0.01), while there was no correlation at 16 weeks (rho = 0.25; *p* = 0.41). Neither WBMRI26 nor US28 correlated with DAS28CRP at baseline (rho = 0.05, *p* = 0.86, and rho = −0.28, *p* = 0.26, respectively) or at week 16 (rho = 0.13, *p* = 0.67; rho = −0.26, *p* = 0.39, respectively).

WBMRI26 did not correlate with TJC28 at baseline (rho = −0.24, *p* = 0.34) nor at week 16 (rho = 0.39, *p* = 0.19). No correlation was found with SJC28 at baseline (rho = 0.37, *p* = 0.13) nor at week 16 (rho = −0.07, *p* = 0.83).

US28 had a negative correlation with TJC28 at baseline and week 16 (rho = −0.53, *p* = 0.02 and rho = −0.36, *p* = 0.23) and no correlation was found for SJC28 at baseline (rho = 0.42, *p* = 0.09) nor at week 16 (rho = 0.23, *p* = 0.46).

### Agreement at Joint Level

**Figure 1:**
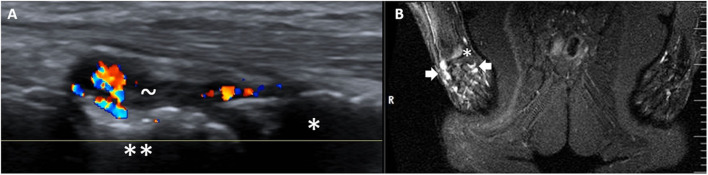
Inflammatory activity in the right (R) wrist as shown by ultrasound **(A)** and WBMRI (STIR) **(B)**. * = radius, ** = schaphoid bone, ~ = synovial hypertrophy with Doppler activity, white thick arrows = high signal intensity in the wrist compatible with inflammation.

In the pooled joint analysis, a moderate agreement was found between WBMRI and ultrasound for the wrist, MCP and PIP joints (κ = 0.41, 0.41, and 0.44, respectively)—Figure 1, whereas the agreement was fair-poor for other joints (κ < 0.40), Table 2.

**Table 2:** Agreement between ultrasound, WBMRI, and clinical evaluation at joint level.

**Sites**	**Ultrasound inflammation**			**MRI inflammation**	
**Kappa*P*-value % agreement *N***	**MRI inflammation**	**Clinically tender joints**	**Clinically swollen joints**	**Clinically tender joints**	**Clinically swollen joints**
Shoulders	0.20	0.10	0.48	0.08	0.05
	0.01	0.247	0.000	0.504	0.485
	80%	81%	97%	67%	72%
	59	59	59	64	64
Elbows	–	0.08	0.37	–	–
		0.514	0.000		
		76%	90%		
		59	59		
Wrists	0.41	0.26	0.13	0.14	−0.01
	0.000	0.037	0.309	0.163	0.908
	69%	64%	57%	52%	42%
	59	61	61	62	62
MCP1–5 joints	0.41	0.10	0.27	0.06	0.07
	0.001	0.362	0.026	0.645	0.955
	70%	52%	62%	54%	51%
	60	61	61	63	63
PIP1–5 joints	0.44	0.22	0.30	0.33	0.36
	0.001	0.067	0.019	0.007	0.003
	76%	62%	74%	67%	74%
	58	61	61	61	61
Knees	0.12	0.15	0.13	0.15	0.07
	0.298	0.198	0.309	0.223	0.433
	61%	69%	80%	60%	58%
	57	59	59	60	60
Ankles	−0.06	0.10	0.18	0.16	0.07
	0.246	0.325	0.110	0.092	0.395
	30%	68%	76%	52%	40%
	61	59	59	62	62
MTP1–5 joints	0.11	0.01	−0.12	0.08	0.08
	0.265	0.918	0.259	0.418	0.102
	59%	53%	41%	63%	35%
	61	59	59	62	62

*Values are kappa (1st row), p-values for kappa statistic (2nd row), Percent agreement (3rd row) and number on observations (n, 4th row)*.

*WBMRI, whole body magnetic resonance imaging; MCP, metacarpophalangeal; PIP, proximal interphalangeal; MTP, metatarsophalangeal*.

The agreement between WBMRI and clinical TJ and SJ was fair-poor with κ < 0.40 for all joints (Table 2).

The agreement between ultrasound and clinical SJC in shoulders was moderate (κ = 0.48), while fair-poor (κ < 0.40) for other joints. Poor agreement was found with TJC (κ < 0.23).

The percent agreement between WBMRI and ultrasound was generally low for ankle, MTP, and knee joints (30, 59, and 6%, respectively) and high for shoulder, MCP, and PIP joints (80, 70, and 76%, respectively).

### Correlation Between Changes During Treatment

After 16 weeks of treatment, median DAS28CRP had decreased from median 4.52 to 3.26, the tender joint count from 7 to 3 and the swollen joint count from 6 to 1, WBMRI26 from 8 to 5 and US28 from 4 to 3—showing a numerical decline for all parameters (Table 1). Six patients (46%) had achieved a good EULAR response, and 7 patients (54%) a moderate response.

The change in WBMRI26 during treatment did not correlate with the change in US28 (rho = 0.38; *p* = 0.21). Neither WBMRI26 nor US28 correlated with the change in DAS28CRP (rho = −0.07, *p* = 0.82 and rho = 0.10, *p* = 0.76, respectively).

## Discussion

This study is the first study to compare whole body assessment of joint inflammation as detected by both WBMRI and ultrasound in clinically active RA patients initiating a biological Disease Modifying anti-rheumatic Drug (DMARD) due to persistent elevated DAS28CRP despite conventional DMARD treatment. We found a good correlation between WBMRI26 and US28 at baseline at patient level, while the agreement at joint level was moderate for the hands and poor for the other joints. Both modalities correlated poorly with the DAS28CRP and clinical joint evaluation.

The strong correlation between WBMRI26 and US28 at baseline (at patient level) combined with the moderate-poor correlation at joint level suggest that ultrasound and MRI both provide measures of the overall inflammatory burden, but take different aspects into account. This could be explained by very different image acquisitions techniques, e.g., ultrasound cannot visualize bone marrow edema. The lack of correlation between the two imaging modalities for changes during treatment and at week 16 may partly be explained by the low level of inflammation in a small patient cohort, particular at follow-up, leaving a narrow disease severity spectrum, which will give small variations in the detected joint inflammation between the two modalities a larger impact on the correlation coefficient. Another contributing factor may be the overall low degree of peripheral inflammation by imaging in the cohort, even at baseline, and hence a lesser potential to improve during treatment.

With the ability to assess multiple joints in a single session ultrasound appeared a well-suited comparator to WBMRI for peripheral inflammatory changes in RA patients. Previous studies comparing conventional MRI and ultrasound have reported a relatively good agreement for synovitis in small peripheral joints (3, 4), i.e., a better agreement than in the present study. When obtaining WBMRI, more anatomical areas are scanned than by conventional MRI and to shorten the imaging time, the image slices are typically thicker and in-plane resolution reduced compared with conventional MRI. Together with the lack of dedicated receiver coils larger voxels and less optimal positioning the image quality is generally lower as compared to conventional MRI with the same field strength. As an example, the hand will at conventional MRI be positioned in a dedicated hand coil in the isocenter of the MRI unit, where the magnetic field is most homogenous, while during WBMRI the hand will have no specific coil and will be positioned below the buttocks, more distant from the isocenter of the magnet. This probably contributed to the observed lower agreement on the individual joint level. It should be emphasized, that the image quality has markedly improved since the study was performed in 2012 and is still undergoing continuous improvements which may positively influence the agreement in the future. Another factor that could have impaired the concordance at joint level is the fact that ultrasound cannot visualize bone marrow edema.

In our study, WBMRI and ultrasound sum scores did not correlate with DAS28CRP at baseline nor at follow-up and the agreement with clinical examination at joint level was generally poor. This is in line with previous studies (22, 23) and may be related to the lower sensitivity of clinical examination for synovitis as compared to ultrasound and MRI (3–6). Furthermore, joint inflammation by imaging was not an inclusion criterion. The low number of patients and the low degree of peripheral inflammation in the investigated cohort may also have contributed to the contra-intuitive findings such as the negative correlation between ultrasound and TJC. WBMRI has the potential, with technical improvements, to become a well-suited tool for clinical trials but is currently not suggested as a clinical tool due to generally lower availability and delay in information to the clinician about the inflammatory status as compared to ultrasound examination.

In conclusion, WBMRI and ultrasound showed good correlation for joint inflammation at patient level indicating that WBMRI is a potential tool for assessing the overall inflammatory burden in RA patients. Further studies implementing recent technical improvements in WBMRI, are needed.

## Data Availability Statement

All datasets generated for this study are included in the article/supplementary material.

## Ethics Statement

The studies involving human participants were reviewed and approved by De Videnskabsetiske Komiteer, Kongens Vænge 2, 3400 Hilleroed, Denmark and the Danish Medicines Agency (EudraCT number NCT01029613). The patients/participants provided their written informed consent to participate in this study.

## Author Contributions

SN has performed data analysis and interpretation and written the manuscript. MA has conducted patient examination, performed data analysis and corrected the proof. MØ has performed data analysis, read WBMRIs, and written the manuscript. SP has performed data interpretation, read WBMRIs, and contributed to the manuscript. IE has read all the WBMRI images and contributed to the manuscript. MH conducted patient examination, contributed to the manuscript. JM has performed all the WBMRIs and contributed to the manuscript. LT has performed all the ultrasound examinations, performed data analysis, and written the manuscript. All authors contributed to the article and approved the submitted version.

## Conflict of Interest

MØ: research support, consultancy fees, and/or speaker fees form AbbVie, BMS, Boehringer-Ingelheim, Celgene, Eli-Lilly, Hospira, Janssen, Merck, Novartis, Novo, Orion, Pfizer, Regeneron, Roche, Sandoz, Sanofi and UCB. SP: speakers fee from MSD, Pfizer, AbbVie, Novartis, and UCB. Advisory board member for AbbVie and Novartis, research support from AbbVie, MSD, and Novartis. MH: funding for research from AbbVie, Biogen, BMS, CellTrion; MSD, Novartis, Orion, Pfizer, Samsung, and UCB. LT: Speakers fee from AbbVie, Janssen, Roche, Novartis, Pfizer, MSD, BMS, and GE. The remaining authors declare that the research was conducted in the absence of any commercial or financial relationships that could be construed as a potential conflict of interest.
